# The surgical time-out: the relationship between perceptions of a safety-task anchor and surgical team workflow

**DOI:** 10.1186/s12893-025-02789-w

**Published:** 2025-02-05

**Authors:** Vivian J. Zagarese, Ivan Hernandez, Neil M. A. Hauenstein, Roseanne J. Foti, Sarah H. Parker

**Affiliations:** 1 Department of Psychology, Virginia Tech, Blacksburg, VA USA; 2 Department of Health Systems and Implementation Science, Virginia Tech Carilion School of Medicine, Roanoke, VA USA; 3 1325 N Pierce St. Apt 102, Arlington, VA 22209 USA

**Keywords:** Time-out, Surgery, Workflow, Teams, Teamwork

## Abstract

**Background:**

The surgical time-out is a critical safety measure used in the operating room (OR). We examined the mediating relationship of the length of the time-out between team perceived usefulness of the time-out, and the rate at which the circulating nurse left the OR to retrieve instruments.

**Methods:**

60 cardiac surgical teams were observed performing their work. The length of the time-out and the rate at which the circulating nurse left the OR was obtained by observation of the surgical team. We administered a survey with a 7-point Likert scale to assess the surgical staff’s perceived usefulness of the time-out at the end of the surgery. An analysis was conducted to test if length of the time-out mediated the relationship between perceived usefulness of the time-out and rate at which the nurse leaves the OR to retrieve an instrument useful for the surgery.

**Results:**

The relationship of the length of the time-out with the rate at which the nurse leaves the OR was non-significant (β = 0.089, *p =* .496). However, the relationship between perceived usefulness of the time-out with the length of the time-out was significant (β = 0.346, *p* < .05) and the effect between perceived usefulness of the time-out and the rate at which the nurse left the OR was statistically significant (β= − 0.424, *p = <* 0.001).

**Conclusion:**

In this study we explore how surgical teams’ attitudes towards the usefulness of the time-out affect its utilization, and how attitudes about time-outs are related to the important process measure of rate at which the circulating nurse leaves the OR. The full mediation model was not supported by the data; however, there appears to be a relationship between the perceived usefulness of the time-out and the rate at which the circulating nurse leaves the OR.

**Supplementary Information:**

The online version contains supplementary material available at 10.1186/s12893-025-02789-w.

## Background

In the United States, approximately 100,000 people die each year due to medical errors, making medical errors a leading cause of death [1]. The Joint Commission, the main accrediting body for hospital quality and safety, has continued to advocate surgical safety as a top priority. To that end, the Joint Commission issues patient safety goals annually; for 2024, “prevent mistakes in surgery” including correct patient, correct site, marking the site, and conducting a pause prior to starting surgery, are an important patient safety goal [2].

Over the last 20 years, researchers, clinicians, and practitioners have developed interventions to prevent surgical mistakes, and particularly to address the foundational issues that contribute to mistakes, such as improving communication and teamwork [3]. Interventions for teams around surgical safety have long included preoperative briefings and other tools to enhance team performance and communication [4–11]. The most widely known intervention during this perioperative period is the World Health Organization (WHO) Surgical Safety Checklist [12, 13].

Within the WHO Surgical Safety Checklist, there are three sub-checklists: one that takes place before the induction of the patient, whereby two independent healthcare professionals confirm the patient’s name, site of surgery, procedure, and consent form. The second “checklist” is the pre-incision pause (we will be referring to it as “time-out”) where the surgical staff pauses and confirms the patient’s name, the procedure, medication, and instruments necessary for the surgical procedure. The final “checklist” is before the patient leaves the OR. At this moment, the surgeon, anesthesiologist, and nurse confirm the count of the instruments, the specimen, and the patient status [1].

A variety of evidence demonstrates that the surgical safety checklist is an effective tool for reducing costs, lowering mortality rates, enhancing the quality of care, improving communication and teamwork dynamics [12–17]. However, some studies have reported mixed results, with limited or no significant changes following the checklist’s implementation [18–20].

Implementation of the checklist has been highly variable, facing numerous barriers [21]. While only a minority of surgical teams use all parts of the WHO checklist, most teams use only the ‘time-out’ portion [22]. Beyond surgical teams not using the full checklist, there are claims that the checklist is often used as a perfunctory checking of a box vs. a meaningful moment to communicate and create shared awareness that negatively affects teamwork, communication of critical patient information, team decision making, delayed procedure start times, and preventable errors [23–27]. Over the last 15 years, most hospitals worldwide have adopted some kind of surgical checklist. From an implementation science perspective, this indicates that the overall rate of checklist adoption has been successful. However, implementation and maintenance require additional inquiry [28]. When implementing checklists, effectiveness is in part a function of accounting for individual perceptions of the usefulness of the implementation [10]. In this paper, we focus on the performance and utilization of the time-out process, acknowledging that these elements contribute to the larger implementation effort. Beliefs and attitudes towards the usefulness of a process that involves information sharing affects the successful implementation of that process [29].

### The role of circulating nurses

Circulating nurses have a critical role when it comes to resource accessibility, equipment malfunctioning, communication, and avoiding extraneous interruptions. They work in the unsterile field of the OR, and they serve in a multifaceted role, they are the patient’s advocate when the patient is under anesthesia and, a source of information about the patient; they help other sterile team members perform their tasks by handing surgical instruments not in the sterile field [30]. Although the most common intraoperative process disruptions are people entering and exiting the OR [31], when circulating nurse leaves the OR to retrieve an instrument that is necessary for the surgical procedure to continue, it can represent a workflow disruption for the surgical team, as well as an increase in potential for airborne pathogens entering the sterile field [32–34].

There are several reasons why a circulating nurse might need to leave the OR during surgery, many of which could be linked to the performance of the time-out. For instance, if the surgical team did not anticipate certain equipment needs during the time-out, this lack of preparation may become apparent once the procedure has begun. Conversely, effective communication during the time-out might identify and resolve potential problems in resources before the open portion of the surgery begins, reducing the likelihood of the nurse needing to leave the OR. The time-out serves as an opportunity to review the standard equipment required and to address any unexpected needs, thereby reducing the frequency of the nurse leaving the OR during the surgery. However, even with a thorough conversation during the time-out, unforeseen events during the procedure, such as equipment malfunction, can result in the circulating nurse leaving the OR.

Better understanding of the impact of the collective perceptions of the usefulness of the time-out procedure is needed. The time-out phase should be more than a perfunctory checklist; rather, it should be a moment for teams to communicate and prepare together. In this study we explore how collective attitudes of the surgical team affect the performance and utilization of the time-out phase, and how these time-out attitudes affect the important process measure of frequencies of circulating nurses leaving the OR.

### Mediational model

**Fig. 1:**
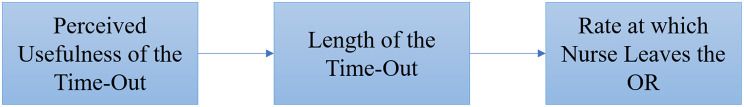
Mediation model. *Note* Length of the time-out (mediator) mediates the relationship between the perceived usefulness of the time-out (independent variable) and the rate at which the nurse leaves the OR (dependent variable)

Our study examined the impact of the collective perceptions of the usefulness of the time-out procedure. More specifically, we explored how participants’ perceptions, as mediated by the length of the time-out phase – which serves as the operational definition of the amount of information exchanged during this phase -- influence the rate at which the circulating nurse leaves the OR. We hypothesized that the relationship between surgical teams’ perceived usefulness of the time-out and the rate at which the circulating nurse leaves the OR is mediated by the length of the time-out. More specifically, we hypothesized that positive perceptions of the usefulness of the time-out will be positively related to the length of the time-out and the length of the time-out will be negatively related to the rate at which the circulating nurse leaves the OR (Fig. 1).

## Methods

This study was approved through the Virginia Tech Institutional Review Board and the Carilion Clinic Institutional Review Board (Carilion Clinic IRB-19-620). The need for consent to participate in this study was waived by the IRB.

### Participants

The participants in this study were 60 cardiovascular surgical teams, from two hospitals. Hospital A – more rural, serving Appalachia and Hospital B – urban, serving a major metropolitan area and surrounding communities. 46 teams were observed at Hospital A, and 14 teams were observed at Hospital B. The teams were usually composed of a surgeon, a fellow or resident or first assistant, one or two anesthesiologists, one or two perfusionists, one or two circulating nurses, and one or two scrub nurses. Team size ranged between six and 10 team members. A total of 11 surgeons were observed (five surgeons from Hospital A and six surgeons from Hospital B). The inclusion criteria for participants were that they had to be surgical team members, specifically working on the surgical case the researcher was observing. Cases that were not open-heart procedures were excluded from the data collection. Not all observed participants completed the questionnaire because some were present only for a brief time during the surgery (e.g., circulating nurses relieving other circulating nurses for lunch breaks). One surgeon, who was observed 10 times, refused to complete the questionnaire and two individuals straight-lined their responses resulting in their data being excluded from the analyses.

The cardiovascular units are usually small, therefore clinical team members were observed multiple times throughout the data collection. The maximum number of times an individual completed the questionnaire was 22. Table 1 shows the total and unique number of participants that completed the questionnaire. Table 2 shows the total and unique participants, by role, that completed the survey. For more information about the overlap of the participants across surgeries please refer to Additional File 1 (Overview of participants and overlap of their presence in multiple surgeries).

**Table 1:** Questionnaire responses by location

Sample	Both locations	A	B
Unique participants that completed the questionnaire	136	77	59
Total participants that completed the questionnaire	457	354	103

**Table 2:** Survey participation by role and response rate to the questionnaire

Professional role	Total questionnaires	Unique participants that completed the questionnaire	Response Rate
Circulating Nurses	97	29	97.9%
Scrub Nurses	78	20	100%
Anesthesiologist	63	28	100%
Perfusionist	71	22	100%
First Assist	36	34	100%
Fellow	10	5	100%
Resident	7	6	100%
Physician Assistant	45	14	100%
Surgeon	50	10	83.3%

### Procedure

The surgical teams were observed from the time-out to wound closure. The anesthesiologist was asked for information regarding the American Society of Anesthesiologists (ASA) status for each patient. ASA status is a standard estimation of how sick the patient is and has been used as an approximate risk score for the surgery in other studies [35]. The ASA score can range from 1 to 6. An ASA of 1 represents a healthy individual; whereas an ASA of 6 represents an individual who is brain dead [36]. At the end of the procedure, all team members were asked to complete the post-operation questionnaire that evaluated their perceived usefulness of the time-out. Additional questions on psychological safety and team trust were also asked in this study but that data was not used for the mediation analysis. Each team member was assigned a unique code which made it possible to count how many unique participants were in the study.

### Measures

#### Perceived usefulness of the time-out

Perceptions were gathered through the questionnaire that was handed to the participants at the end of the surgical procedure. There are no validated questionnaires in the literature that assess this construct; therefore, we used two questions (question 9 and 10 in Additional File 2: Post-Operation Questionnaire) to gather the perceived usefulness of the time-out on a 7-point Likert Scale. To provide evidence of content validity we calculated the Pearson correlation between the two items aggregated at the team level (*r* = .626, *p* < .001) which showed a moderate correlation.

#### Length of the time-out

The length of the time-out was measured from the time when the surgeon or the circulating nurse started the time-out by saying “let’s time-out” (or similar) and ended with the final introduction of the last team member (also the last step on the time-out checklist). Length was recorded through an automatic time stamp in the note taking app used by the researcher. The researcher present in the room recorded all interactions during the time-out. Although this measure does not directly assess the quality of the time-out — since we did not record if the team went over every checklist point in detail—the length of the time-out gives an indication of how it was performed: a 10 s time-out has less time for information exchange than a one-minute time-out.

#### Rate at which the circulating nurse leaves the OR

Circulating nurses are essential in ensuring the availability of resources, addressing equipment issues, supporting teamwork, facilitating communication, and minimizing unnecessary interruptions. When circulating nurses leave the OR to retrieve an instrument not discussed during the time-out, their duties related to patient care are suspended, potentially causing workflow disruptions that increase the time the patient is under bypass [37]. The researcher present in the OR recorded every time the circulating nurse left the OR to retrieve an instrument that was necessary for a successful surgery. The times the circulating nurse left for a break and the times the circulating nurse left with an orientee nurse for educational purposes were not recorded. Although such departures can be disruptive, they are also not likely to be directly affected by the length of the time-out.

#### Control variables

Length of surgery, hospital location, and surgeon were included as control variables in the analyses. Some surgeons naturally differ in the way they conduct the time-out process. For example, certain surgeons may take longer time-outs on average than others, due to differences in their routines or approach to team communication. To account for this potential source of variation, the individual surgeon was considered a control variable in the analysis. Although ASA scores of patient criticality were available, they were not included in the analyses because all patients had a score of 4, which indicates the presence of severe systemic disease that is a constant threat to life. This was reflective of the high-risk nature of the cases observed, particularly in the cardiac surgery setting.

#### Contextual variables

Data on psychological safety and team trust was gathered during the course of the study with the post-operation questionnaire. Although these variables are not included in the proposed mediation model, they provide valuable insight on the contextual factors that could influence the process measures of the model. Additional File 3 (Additional Contextual Variables: Psychological Safety and Team Trust) includes information on these variables as well as information on when the Surgical Safety Checklist was implemented in the data collection sites.

## Results

### Power analysis

An a priori power analysis was conducted using a one tailed test for correlation. The number of teams needed for a medium effect size (0.30), a power of 0.75 and an alpha = 0.05, is 59. Although sixty teams were observed, a boxplot of the perceived usefulness of the time-out revealed that two teams were outliers; therefore, all analysis was conducted with 58 teams.

### Data preparation

#### Aggregation of the individual perceptions of the time-out usefulness to team-level variable

Perceptions of time-out usefulness were assessed using a post-operation questionnaire given to all team members. To compute individual perceptions of time-out usefulness, responses to the two usefulness questions were averaged. Surgical team-level perceptions of time-out usefulness was computed by averaging each team members’ individual perceptions of time-out usefulness. It is generally recognized that computing team-level variables by aggregating individual perceptions requires further evidence of strong interrater agreement among team members. When using shared emergent states, the most common operational definition of interrater agreement is the r_wg_ index [38]. However, given surgical teams represent different types of expertise, (e.g., anesthesia, nursing etc.), strict adherence to the traditional r_wg_ of at least 0.7 may be too stringent [39].

Alternative approaches, such as using confidence intervals (CIs) allow to incorporate uncertainty rather than a single point cutoff threshold [40]. However, CIs also have limitations because they can be affected by the underlying diversity of the team. Given the debate, we proceed with the analyses for researchers wanting to know the agreement among the different teams. We have included Additional File 4 (Additional Contextual Variables: Psychological Safety and Team Trust) that presents the r_wg_ results alongside their corresponding confidence intervals.

#### Time-out length and frequency of circulating nurse leaving the OR

The time-out length was retrieved by the time stamps in the excel sheet of the live coding. This measure was reported in seconds. The frequency of the circulating nurse leaving the OR was counted from the excel sheet of the coder that reported this information during the live coding. Since the surgeries were of varying length, the frequency by which the circulating nurse left the OR was higher when the surgeries were longer. To control for this phenomenon, we divided the number of times the circulating nurse left the OR by the length (in minutes) of the surgery. In all future analysis the rate of departure per minute was used.

### Control analysis

This study is a field study; therefore, we were unable to manipulate the individuals staffing the OR in our research design. We conducted a control analysis to determine which variables should be adjusted based on the surgeon and location. Control variables were tested for inclusion, but not included in all analysis. For more details on how the control analysis was conducted see Additional File 5 (Control Analysis). Based on the results of the control analysis, time-out length was adjusted for both surgeon and location.

The descriptive statistics (Table 3) and the correlation matrix (Table 4) report both the unadjusted and the adjusted measures of the variables used in this study.

**Table 3:** Descriptive statistics

Variable	*N*	Minimum	Maximum	Mean	St. Dev.
Perception of the time-out usefulness	58	4.939	6.940	5.852	0.467
Time-out length (seconds, original measure)	58	14.00	127.00	61.431	28.080
Time-out length (adjusted for location and surgeon)	58	-2.60	2.34	0.00	0.991
Nurse leaves OR (original measure)	58	2	17	7.655	3.620
Nurse leaves OR (adjusted for length of surgery)	58	0.010	0.070	0.033	0.013
Surgery length (minutes)	58	81.00	380.00	229	64.145

*Note* Table 3 presents the descriptive statistics of the variables used in the study, aggregated at the team level. A total of 457 questionnaires were collected and aggregated into scores for 58 surgical teams. Scores for the perception of the usefulness of the time-out were obtained using a 7-point Likert scale, where 1 indicated “very inaccurate” and 7 indicated “very accurate.” Adjusted measures (e.g., time-out length) were calculated to control for variables such as surgeon and hospital location. By adjusting the variable, the mean became zero which is why some values may appear as negative

**Table 4:** Correlation matrix of original and adjusted variables

Variable	1.	2.	3.	4.	5.
1. Perceptions of the time-out					
2. Time-out length	0.616**				
3. Time-out length (adjusted)	0.346**	0.619**			
4. Nurse leaves OR	− 0.413**	− 0.439**	− 0.015		
5. Nurse leaves OR (adjusted)	− 0.424**	− 0.337**	− 0.068	0.827**	
6. Surgery length	− 0.180	− 0.376**	− 0.059	0.586**	0.070

** Correlation is significant at the 0.01 level (two-tailed)

Table 4 presents correlations between the original and adjusted variables to illustrate the relationships among perceptions of the time-out, time-out length, the rate at which the nurse leaves the OR, and surgery length. The significance levels (*p* < .01) highlight which relationships are statistically meaningful.

The variable perception of the usefulness of the time-out is significantly correlated with the time-out length (*r* = .616, *p* < .001). This suggests that as the time-out is perceived as more useful, teams take more time performing it. After adjustment the correlation decreases but remains positive and statistically significant (*r* = .346, *p* < .001). This adjustment accounts for the surgeon and location of the surgery.

A moderate negative correlation between time-out length and frequency of nurse leaving the OR (*r*=-.439, *p* < .001) suggests that longer time-outs are associated with the nurse leaving the OR at a lower rate.

A moderately strong negative correlation between the perceptions of the time-out and the frequency of nurse leaving the OR (*r* = − .413, *p* < .001) suggests that as the perceptions of the time-out improve, the rate of the nurse leaving the OR decreases. The relationship remains robust even after controlling for the length of the surgery (*r* = − .424, *p* < .001).

### Mediation analysis

**Fig. 2:**
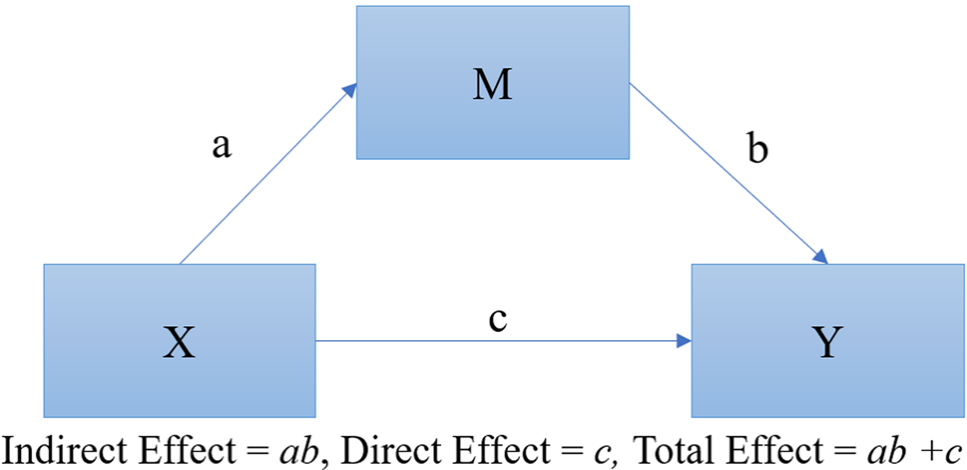
General mediation model where path coefficient “c” represents the direct effect of the independent variable (X) on the dependent variable (Y) and path coefficients “a” and “b” represent the indirect effect of the mediating variable (M) on Y

A variable is considered a mediator when it explains part or all of the relationship between an independent variable and a dependent variable. Figure 2 depicts the general mediation model as depicted from Baron and Kenny [41] showing both the direct effect of the independent variable on the dependent variable and the indirect effects of the mediating variable on the relationship between the independent variable and the dependent variable.

### Model results

Mediating analyses recommended by Baron and Kenny [41] were used to test the hypothesis that the relationship between team-level perceptions of time-out usefulness and frequency of the circulating nurse leaving the OR is explained by length of the time-out. First, the total effect of perceptions of the time-out usefulness and the rate at which the nurse left the OR (adjusted for length of surgery) was tested using a bivariate regression (Fig. 2). Standardized βs were used to report the effects to compare the effect size more easily. The results show that there is a statistically significant negative effect between perceived usefulness of the time-out and the rate at which the circulating nurse leaves the OR (β = − 0.424, *p* < .001, Std. Err = 0.003, t = -3.501, R^2^ = 0.179, df = 57).

Next, the effect (*a)* of perceived usefulness of the time-out and length of the time-out (controlling for surgeon and location), was again estimated using a bivariate regression. A significant effect between the perceptions of the time-out usefulness and the length of the time-out was found (β = 0.346, *p* < .05, Std. Err = 0.241, t = 2.759, R^2^ = 0.120, df = 57).

**Fig. 3:**
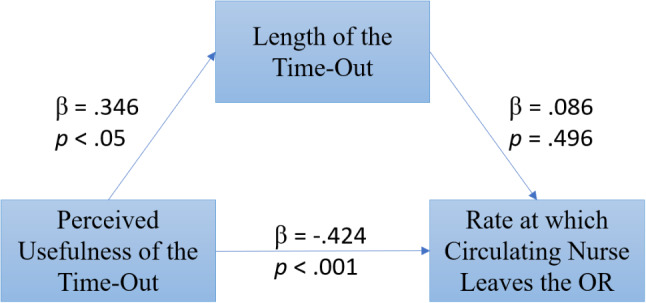
Overall mediation model with standardized beta weights and *p*-values. *Note* Length of the time-out (mediator) mediates the relationship between the perceived usefulness of the time-out (independent variable) and the rate at which the nurse leaves the OR (dependent variable)

The direct effect between perceived usefulness of the time-out and the rate at which the nurse left the OR was estimated with multiple regression: perceived usefulness of the time-out and length of the time-out used as predictors and rate at which the circulating nurse left the OR as the dependent variable. The estimation of the direct effect of perceived usefulness of the time-out and length of the time-out on nurse leaving the OR was non-significant (β = 0.089, *p* = .496, Std. Err = 0.002, t = 0.686, R^2^ = 0.186, df = 57). The results of the mediation model are portrayed in Fig. 3.

Lastly, the Sobel test was used to estimate the indirect effect and tested for statistical significance using a z-test statistic. The Sobel test was non-significant with a test statistic of 0.249, a standard error of 0.002 and a p-value of 0.803. While there is support for the direct effect of the perception of the time-out affecting the rate at which the circulator nurse left the OR, there was no statistical support for the length of the time-out mediating the perceived usefulness of the time-out and the frequency with which the circulator nurse left the OR.

### Exploratory model

Upon further examination, there was a potential issue about the shared variance between question 10 that directly asked respondents to assess their perceived usefulness of the time-out for the specific operation, and the dependent variable (frequency of nurse leaving the OR). The Pearson correlation between the two items aggregated at the team level *r* = .626 (*p* < .001) showed a moderate correlation. The shared variance may have limited the item’s ability to reflect a general attitude towards time-outs and affect its role as a mediator. Consequently, we tested an exploratory mediation model where the length of the time-out serves as the independent variable, the perceived usefulness of the time-out is the mediator, and the rate at which the nurse leaving the OR is the dependent variable.

The analysis was conducted by estimating the effect between the length of the time-out and the rate at which the nurse left the OR with a bivariate regression. The results show that there is a non-significant negative effect between perceived usefulness of the time-out and the rate at which the circulating nurse leaves the OR (β = − 0.068, *p* = .610, Std. Err = 0.002, t = − 0.513, R^2^ = 0.004, df = 57).

The effect (*a*) between the length of the time-out and the mediator, perceived usefulness of the time-out, was then estimated using a bivariate regression. The results showed a significant effect of the length of the time-out and perceived usefulness of the time-out (β = 0.346, *p* < .05, Std. Err = 0.065, t = 2.759, R^2^ = 0.120, df = 57).

**Fig. 4:**
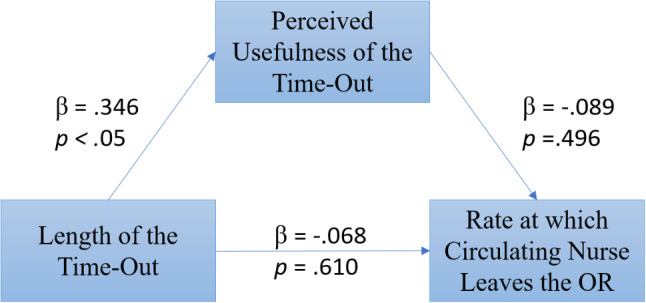
Overall model with standardized beta weights and *p*-values. *Note* Perceived usefulness of the time-out (mediator) mediates the relationship between the length of the time-out (independent variable) and the rate at which the nurse leaves the OR (dependent variable)

The direct effect between length of the time-out and the rate at which the nurse left the OR was estimated with multiple regression: length of the time-out and perceived usefulness of the time-out used as predictors and rate at which the circulating nurse left the OR as the dependent variable. The estimation of the direct effect of length of the time-out and perceived usefulness of the time-out on nurse leaving the OR was non-significant (β = 0.089, *p* = .496, Std. Err = 0.002, t = 0.686, R^2^ = 0.120, df = 57). The results of the exploratory mediation model are portrayed in Fig. 4.

Lastly, the Sobel test was used to estimate the indirect effect and tested for statistical significance using a z-test statistic. The Sobel test was non-significant with a test statistic of -0.492, a standard error of 0.001 and a p-value of 0.622.

The exploratory mediation analysis did not meet the prerequisites for mediation, as the direct effect of time-out length on nurse exit frequency was non-significant. However, the model provided useful insights into the relationship between the length and the perceived usefulness of the time-out.

## Discussion

In the model analysis we examined one safety critical task, the time-out, and team perceptions of time-out value, and its relationship on one process measure of safety—rate at which the circulator nurse leaves the OR. For the original hypothesis, results failed to support the expected partial mediation model because the relationship of the length of the time-out with the rate at which the nurse leaves the OR (path b) was non-significant. However, the relationship between perceived usefulness of the time-out with the length of the time-out (path a) and the relationship of the perceived usefulness of the time-out and the rate at which the nurse left the OR was statistically significant. These findings suggest that the perceived usefulness of the time-out matters because it decreases the time the circulating nurse is absent to receive information and relay it to other team members. Furthermore, the absence of a device/instrument in the room increases the time that the surgical team is waiting for it, putting the patient under longer bypass times and increased risks of infection.

Previous research has highlighted the importance of balanced communication in high-performing teams, and the role of thorough time-out execution in ensuring team preparedness [15]. However, evidence also indicates variability in adherence to the protocol, with some teams adopting a “tick and flick” approach [42–44].

The results align with evidence that team attitudes toward safety protocols can influence their performance and outcomes [29]. When team members view the time-out as a meaningful safety measure, it may lead to behaviors that minimize disruptions and support patient care. Conversely, non-compliance may contribute to a poorer outcome [21].

The findings of this study may provide the opportunity for organizational leaders to remind surgical teams of the value of the standard protocol, emphasizing that it serves as more than a perfunctory checklist and encouraging all team members to actively engage in safety practices.

The hypothesized model was based on the premise that the perceived usefulness of a protocol can shape behavior when using that protocol. However, it is important to acknowledge that this is just one of several possible models. The length of the time-out might influence the perceived usefulness of the protocol which could then impact the frequency with which the circulating nurse leaves the OR. Another alternative is that the length of the time-out moderates the relationship between the perceived usefulness of the time-out and the rate at which the circulating nurse leaves the OR.

There are several limitations to this study. First, there are no existing validated scales that measure perceptions of time-out value. We used two questions because the surgical staff had limited time to complete the questionnaires. It is possible that we are missing important additional aspects of the perceived usefulness of the time-out. Although the time-out checklist contained an item about the instruments in both hospitals, the observer did not record whether this specific item was discussed during the time-out. If the surgical team did not discuss the instruments, our model might reflect a causal mechanism related to the omission of this discussion rather than the perceptions of the usefulness of the time-out. Ideally, future work would consider a nuanced and detailed analysis of the time-out by identifying which items were included and the relationship between these items and their impact on surgical care. Given the length of most timeouts, it is very difficult to record all that information reliably without a video or audio recording, which would introduce complex confidentiality considerations, potentially minimizing the sample. Question 10 evaluates the perceived usefulness of the time-out for the specific operations, and the wording may partially overlap with the dependent variable (frequency of nurse leaving the OR). To address this potential overlap in the constructs, we tested the exploratory model. We also assume that longer time-outs indicate higher quality. While it is true that a 10 s time-out allows less time for information exchange than a one-minute time-out, it does not necessarily mean that the longer time-out was more valuable. Another limitation is represented by the fact that the perceived usefulness of the time-out was measured with the post-operation questionnaire once the surgery was completed. It would not have been feasible for the surgical team to complete the questionnaire right after the time-out as surgical team members are scrubbed in and it would have represented a substantial workflow disruption.

A possible unexplained variable that could have affected the variables in this study is the organizational safety culture of the hospital. When safety and quality officers do random quality checks of the time-out, it may send the message to the staff that performing the full time-out is important to the hospital, thereby changing the attitude of the team towards the time-out and its outcomes. We were not able to control for this at the hospitals we recruited for participation. Future studies should attempt to better understand the macro-level organizational culture. Another possible unexplained variable is the time of the day in which the surgery starts. If there are delays, the surgical staff could become impatient and the length of the time-out and the rate at which the circulating nurse leaves the OR could be affected. A body of literature (e.g. [45, 46]) focuses on safety events, and particularly errors in relation to perioperative processes such as teamwork. We did not include errors in the data due to the difficulty in identifying them without clinical expertise. Future studies should consider exploring non-routine events in relation to the compliance and perceptions of the usefulness of the time-out.

Another limitation of this study is the aggregation of the responses to the team level for the perceived usefulness of the time-out. Since different roles within the surgical team might have varying perspectives on the time-out and given that there were more responses from nurses than from surgeons, this could have led to a stronger nursing perspective. Furthermore, the cardiovascular units are usually small, therefore clinical team members were observed multiple times throughout the data collection which may lead to non-independent data.

Mitchell and colleagues [47] emphasize the importance of evaluating how research on the checklist is conducted. They point out the critical need to take into account the contextual factors in this research. In this study, we used controlling contextual variables for the surgeon, length of surgery, and hospital location; however more specific variables that could affect the process of the time-out, such as teamwork and communication, as well as organizational elements like safety culture, and the ambiguities that often accompany checklist implementation may provide insight on the true value of this tool.

## Conclusion

In this study we showed that surgical teams who have a positive perception of the usefulness of the time-out achieve better performance as operationalized by the rate at which the circulating nurse leaves the OR.

The findings suggest that enhancing team buy-in and attitudes toward the time-out process could mitigate workflow interruptions and contribute to safer surgical environments. The mechanism by which this happens is still unclear, given that the mediation model was not supported and unmeasured variables may have influenced the outcome measure.

Future research should explore additional mediators and contextual factors, such as organizational culture and team dynamics, to better understand the mechanisms underlying these relationships.

## Electronic supplementary material

Below is the link to the electronic supplementary material.

Supplementary Material 1

Supplementary Material 2

Supplementary Material 3

Supplementary Material 4

Supplementary Material 5

## Data Availability

The data used and analyzed during the current study are available from the corresponding author on reasonable request.
