# Supplementary material for: The surgical time-out: the relationship between perceptions of a safety-task anchor and surgical team workflow
Source: BMC Surg. 2025 Feb 5;25:55. doi: 10.1186/s12893-025-02789-w (PMC11796080; doi:10.1186/s12893-025-02789-w)
Supplement: Supplementary file 1 — Supplementary Material 1 [file 12893_2025_2789_MOESM1_ESM.docx]

**Additional file 1**

**Overview of participants and overlap of their presence in multiple surgeries**

Table S1 shows how many times each participant was present in a surgery (e.g., participant 18 was present in 22 surgeries: 17 times as a circulating nurse and 5 times as a scrub nurse).

**Table S1**

*Overview of participants and their presence across surgeries*

| Participant | Circulating nurse | Scrub nurse | Anesthesiologist | Perfusionist | First Assist | Fellow | Resident | Physician Assistant | Surgeon | Total by participant |
| --- | --- | --- | --- | --- | --- | --- | --- | --- | --- | --- |
| 1 |  |  |  |  |  |  |  |  | 10 | 10 |
| 2 |  |  |  |  |  |  |  |  | 15 | 15 |
| 3 |  |  |  |  |  |  |  |  | 5 | 5 |
| 4 |  |  |  |  |  |  |  |  | 6 | 6 |
| 5 |  |  |  |  |  |  |  |  | 3 | 3 |
| 6 |  |  |  |  |  |  |  |  | 3 | 3 |
| 7 |  |  |  |  |  |  |  |  | 2 | 2 |
| 8 |  |  |  |  |  |  |  |  | 2 | 2 |
| 9 |  |  |  |  |  |  |  |  | 2 | 2 |
| 10 |  |  |  |  |  |  |  |  | 2 | 2 |
| 11 | 1 |  |  |  |  |  |  |  |  | 1 |
| 12 |  | 2 |  |  |  |  |  |  |  | 2 |
| 13 |  |  | 1 |  |  |  |  |  |  | 1 |
| 14 |  | 1 |  |  |  |  |  |  |  | 1 |
| 15 | 1 |  |  |  |  |  |  |  |  | 1 |
| 16 | 1 |  |  |  |  |  |  |  |  | 1 |
| 17 |  |  |  | 6 |  |  |  |  |  | 6 |
| 18 | 17 | 5 |  |  |  |  |  |  |  | 22 |
| 19 |  |  |  | 2 |  |  |  |  |  | 2 |
| 20 | 1 | 2 |  |  | 6 |  |  |  |  | 9 |
| 21 |  |  | 3 |  |  |  |  |  |  | 3 |
| 22 |  |  |  |  |  |  |  | 1 |  | 1 |
| 23 | 5 | 2 |  |  |  |  |  |  |  | 7 |
| 24 | 15 |  |  |  |  |  |  |  |  | 15 |
| 25 |  |  |  |  |  | 2 |  |  |  | 2 |
| 26 | 13 | 3 |  |  |  |  |  |  |  | 16 |
| 27 | 1 | 2 |  |  |  |  |  |  |  | 3 |
| 28 |  |  | 8 |  |  |  |  |  |  | 8 |
| 29 |  |  |  | 1 |  |  |  |  |  | 1 |
| 30 |  | 2 |  |  |  |  |  |  |  | 2 |
| 31 |  |  | 3 |  |  |  |  |  |  | 3 |
| 32 |  |  |  | 10 |  |  |  |  |  | 10 |
| 33 |  |  |  |  |  |  | 1 |  |  | 1 |
| 34 |  |  | 1 |  |  |  |  |  |  | 1 |
| 35 |  |  | 2 |  |  |  |  |  |  | 2 |
| 36 |  |  |  |  |  |  | 2 |  |  | 2 |
| 37 |  |  |  | 2 |  |  |  |  |  | 2 |
| 38 |  |  |  | 1 |  |  |  |  |  | 1 |
| 39 |  | 1 |  |  |  |  |  |  |  | 1 |
| 40 |  |  | 6 |  |  |  |  |  |  | 6 |
| 41 |  |  |  | 1 |  |  |  |  |  | 1 |
| 42 |  | 2 |  |  |  |  |  |  |  | 2 |
| 43 | 1 |  |  |  |  |  |  |  |  | 1 |
| 44 |  |  |  |  |  | 4 |  |  |  | 4 |
| 45 |  |  |  |  |  | 1 |  |  |  | 1 |
| 46 | 2 |  |  |  |  |  |  |  |  | 2 |
| 47 | 5 | 4 |  |  | 4 |  |  |  |  | 13 |
| 48 |  |  |  |  | 1 |  |  |  |  | 1 |
| 49 | 2 |  |  |  |  |  |  |  |  | 2 |
| 50 |  |  |  | 6 |  |  |  |  |  | 6 |
| 51 |  |  | 2 |  |  |  |  |  |  | 2 |
| 52 | 1 |  |  |  |  |  |  |  |  | 1 |
| 53 |  |  |  |  |  |  | 1 |  |  | 1 |
| 54 |  |  | 1 |  |  |  |  |  |  | 1 |
| 55 |  |  | 1 |  |  |  |  |  |  | 1 |
| 56 |  |  |  | 2 |  |  |  |  |  | 2 |
| 57 |  | 1 |  |  |  |  |  |  |  | 1 |
| 58 |  |  | 1 |  |  |  |  |  |  | 1 |
| 59 |  |  | 1 |  |  |  |  |  |  | 1 |
| 60 | 1 | 5 |  |  | 2 |  |  |  |  | 8 |
| 61 | 1 | 5 |  |  | 4 |  |  |  |  | 10 |
| 62 |  |  |  |  |  | 1 |  |  |  | 1 |
| 63 |  |  |  | 2 |  |  |  |  |  | 2 |
| 64 |  |  | 1 |  |  |  |  |  |  | 1 |
| 65 |  |  |  |  |  |  |  | 1 |  | 1 |
| 66 |  |  |  | 1 |  |  |  |  |  | 1 |
| 67 | 2 |  |  |  |  |  |  |  |  | 2 |
| 68 |  |  |  |  |  |  |  | 2 |  | 2 |
| 69 |  |  |  |  |  |  |  | 3 |  | 3 |
| 70 |  |  |  | 6 |  |  |  |  |  | 6 |
| 71 |  | 1 |  |  |  |  |  |  |  | 1 |
| 72 |  |  | 1 |  |  |  |  |  |  | 1 |
| 73 |  |  |  |  |  |  |  | 1 |  | 1 |
| 74 | 1 |  |  |  |  |  |  |  |  | 1 |
| 75 |  |  |  |  |  |  |  | 10 |  | 10 |
| 76 |  |  |  | 1 |  |  |  |  |  | 1 |
| 77 |  | 3 |  |  |  |  |  |  |  | 3 |
| 78 | 4 |  |  |  |  |  |  |  |  | 4 |
| 79 |  | 3 |  |  |  |  |  |  |  | 3 |
| 80 |  | 4 |  |  | 10 |  |  |  |  | 14 |
| 81 |  |  | 3 |  |  |  |  |  |  | 3 |
| 82 |  | 3 |  |  | 1 |  |  | 1 |  | 5 |
| 83 |  |  |  | 2 |  |  |  |  |  | 2 |
| 84 |  |  |  | 3 |  |  |  |  |  | 3 |
| 85 |  |  | 8 |  |  |  |  |  |  | 8 |
| 86 |  | 1 |  |  |  |  |  |  |  | 1 |
| 87 |  |  |  |  |  |  |  | 10 |  | 10 |
| 88 |  | 3 |  |  |  |  |  |  |  | 3 |
| 89 |  |  |  |  |  |  |  | 11 |  | 11 |
| 90 | 2 |  |  |  |  |  |  |  |  | 2 |
| 91 | 5 |  |  |  |  |  |  |  |  | 5 |
| 92 | 1 |  |  |  |  |  |  |  |  | 1 |
| 93 |  |  | 1 |  |  |  |  |  |  | 1 |
| 94 | 1 |  |  |  |  |  |  |  |  | 1 |
| 95 |  |  | 4 |  |  |  |  |  |  | 4 |
| 96 | 4 |  |  |  |  |  |  |  |  | 4 |
| 97 |  |  | 1 |  |  |  |  |  |  | 1 |
| 98 |  |  | 2 |  |  |  |  |  |  | 2 |
| 99 | 3 |  |  |  |  |  |  |  |  | 3 |
| 100 | 3 | 10 |  |  | 2 |  |  |  |  | 15 |
| 101 |  | 7 |  |  | 4 |  |  |  |  | 11 |
| 102 |  |  | 2 |  |  |  |  |  |  | 2 |
| 103 |  |  | 1 |  |  |  |  |  |  | 1 |
| 104 |  |  | 1 |  |  |  |  |  |  | 1 |
| 105 |  |  | 1 |  |  |  |  |  |  | 1 |
| 106 |  |  |  | 1 |  |  |  |  |  | 1 |
| 107 |  |  | 4 |  |  |  |  |  |  | 4 |
| 108 |  |  |  |  |  | 2 |  |  |  | 2 |
| 109 |  |  | 1 |  |  |  |  |  |  | 1 |
| 110 |  |  |  |  |  |  | 1 |  |  | 1 |
| 111 |  |  |  | 1 |  |  |  |  |  | 1 |
| 112 | 1 |  |  |  |  |  |  |  |  | 1 |
| 113 |  |  |  | 10 |  |  |  |  |  | 10 |
| 114 |  |  | 1 |  |  |  |  |  |  | 1 |
| 115 |  |  |  | 9 |  |  |  |  |  | 9 |
| 116 |  |  |  |  |  |  | 1 |  |  | 1 |
| 117 |  | 1 |  |  |  |  |  |  |  | 1 |
| 118 |  |  |  | 2 |  |  |  |  |  | 2 |
| 119 |  | 1 |  |  |  |  |  |  |  | 1 |
| 120 |  |  |  |  |  |  |  | 1 |  | 1 |
| 121 |  |  |  | 1 |  |  |  |  |  | 1 |
| 122 |  |  | 1 |  |  |  |  |  |  | 1 |
| 123 |  |  |  |  |  |  |  | 1 |  | 1 |
| 124 |  |  |  |  |  |  | 1 |  |  | 1 |
| 125 |  | 1 |  |  |  |  |  |  |  | 1 |
| 126 |  | 1 |  |  |  |  |  |  |  | 1 |
| 127 | 1 |  |  |  |  |  |  |  |  | 1 |
| 128 | 1 |  |  |  |  |  |  |  |  | 1 |
| 129 |  |  |  | 1 |  |  |  |  |  | 1 |
| 130 |  |  |  |  |  |  |  | 1 |  | 1 |
| 131 |  | 1 |  |  |  |  |  |  |  | 1 |
| 132 |  | 1 |  |  |  |  |  |  |  | 1 |
| 133 |  |  |  |  |  |  |  | 1 |  | 1 |
| 134 |  |  |  |  |  |  |  | 1 |  | 1 |
| 135 |  |  |  |  | 1 |  |  |  |  | 1 |
| 136 |  |  |  |  | 1 |  |  |  |  | 1 |
| Total by role | 97 | 78 | 63 | 71 | 36 | 10 | 7 | 45 | 50 | 457 |
