# Supplementary figures and images for: The surgical time-out: the relationship between perceptions of a safety-task anchor and surgical team workflow
Source: BMC Surg. 2025 Feb 5;25:55. doi: 10.1186/s12893-025-02789-w (PMC11796080; doi:10.1186/s12893-025-02789-w)

**Additional File 2**

**Post-Operation Questionnaire**


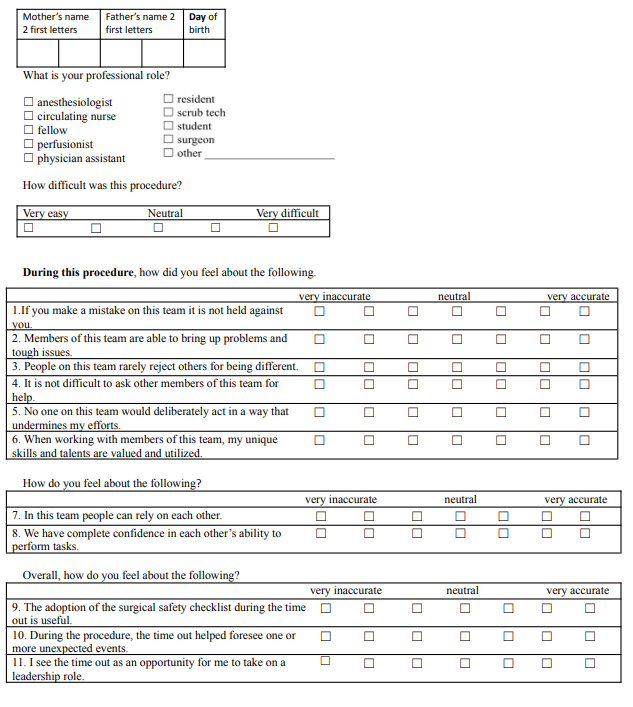

Supplement: Supplementary file 2 — Supplementary Material 2 [file 12893_2025_2789_MOESM2_ESM.docx]
