# Supplementary material for: The surgical time-out: the relationship between perceptions of a safety-task anchor and surgical team workflow
Source: BMC Surg. 2025 Feb 5;25:55. doi: 10.1186/s12893-025-02789-w (PMC11796080; doi:10.1186/s12893-025-02789-w)
Supplement: Supplementary file 3 — Supplementary Material 3 [file 12893_2025_2789_MOESM3_ESM.docx]

**Additional File 3**

**Additional Contextual Variables: Psychological Safety and Team Trust**

Additional contextual variables may give insight into the findings of our study. We provide information on two team level variables – psychological safety and team trust—and on the implementation of the Surgical Safety Checklist in the data collection sites.

**Implementation of the Checklist**

In 2003 Hospital A launched time-outs as part of the Universal Protocol for the prevention of wrong procedure/site/patient. Since then, the checklist has grown from a very simplistic 2-3 item list performed right before incision to the three-part World Health Organization (WHO) Surgical Safety Checklist. Hospital B was part of the WHO Surgical Safety Checklist pilot study and had the checklist implemented in 2007.

**Psychological Safety and Team Trust**

Psychological safety and team trust are two variables that were collected as a part of a larger study. We report these variables here to offer additional insight into contextual variables that may have affected the findings of the study.

Psychological safety was measured with the scale created by Edmondson (46). The original scale contains seven questions. One of the questions – “is it safe to take risks on this team”- as pointed out by several surgeons, is non-specific and up to interpretation. Taking a risk can be interpreted as a social risk (i.e., speaking up about something wrong that someone has done) or as a risk involving the patient or the task at hand (i.e., trying a new surgical method). Due to this ambiguity, the question was not included in the questionnaire, therefore psychological safety was assessed with the remaining six questions. The psychological safety measure shows good internal consistency with a Cronbach’s alpha of .82 (48). Psychological safety was measured with a 7-point Likert scale, where 1 corresponds with “very inaccurate”, 4 with “neutral” and 7 with “very accurate”. Some of the questions were framed in a negative way (i.e., “If you make a mistake on this team, it is often held against you”). The surgical staff, due to time constraints, sometimes does not read the questions fully, therefore the negatively framed questions were all reversed in the positive (i.e., “if you make a mistake on this team, it is *not* held against you”). This was done to avoid errors in the responses.

**Team Trust.** The Costa and Anderson (2011) scale of trust was used (49). This scale was tested in two different samples: 98 hospital professionals and 395 social-care professionals. The scale is made up of 4 subscales (propensity to trust; perceived trustworthiness; cooperative behaviors; monitoring behaviors) with a total of 21 items. Given the time limit for surgical team members to complete the questionnaire, only the perceived trustworthiness scale was chosen. This scale has six questions with a Cronbach’s alpha of .88. Due to the time constraints in filling out the questionnaire only 2 questions were asked from the perceived trustworthiness subscale: “in this team people can rely on each other”; “we have complete confidence in each other’s ability to perform tasks”. The first question was chosen because of the interdisciplinary nature of the team. Surgical team members need to rely on each other to receive information that is not in their professional field (i.e., the nurse needs to rely on the anesthesiologist to communicate accurate information that only the anesthesiologist has access to). The second question was asked because surgery is task based. Team trust was measured with a 7-point Likert scale, where 1 corresponds with “very inaccurate”, 4 with “neutral” and 7 with “very accurate”.

Table S2 provides the descriptive statistics of psychological safety and team trust. Table S3 provides a correlation matrix of the study variables with the psychological safety and team trust variables.

**Table S2**

*Descriptive Statistics of Psychological Safety and Team Trust*

| Variable | N | Minimum | Maximum | Mean | St. Dev. |
| --- | --- | --- | --- | --- | --- |
| Psychological Safety | 58 | 3.214 | 6.833 | 5.797 | .599 |
| Team Trust | 58 | 4.00 | 6.833 | 6.025 | .525 |

**Table S3**

*Correlation Matrix of Study Variable and Psychological Safety and Team Trust*

| Variable | 1. | 2. | 3. | 4. | 5. | 6. | 7. | 8. |
| --- | --- | --- | --- | --- | --- | --- | --- | --- |
| 1. Perceptions of the time-out |  |  |  |  |  |  |  |  |
| 2. Time-out length | .616** |  |  |  |  |  |  |  |
| 3. Time-out length (adjusted) | .346** | .619** |  |  |  |  |  |  |
| 4. Nurse leaves OR | -.413** | -.439** | -.015 |  |  |  |  |  |
| 5. Nurse leaves OR (adjusted) | -.424** | -.337** | -.068 | .827** |  |  |  |  |
| 6. Surgery length | -.180 | -.376** | -.059 | .586** | .070 |  |  |  |
| 7. Psychological Safety | .199 | .161 | .005 | .018 | .082 | -.124 |  |  |
| 8. Team Trust | .223 | .078 | .001 | .002 | -.015 | .019 | .704** |  |

The correlation matrix shows that psychological safety and team trust have a strong relationship; however, they appear to not be related to the variables used in the mediation model.
