# Supplementary material for: The surgical time-out: the relationship between perceptions of a safety-task anchor and surgical team workflow
Source: BMC Surg. 2025 Feb 5;25:55. doi: 10.1186/s12893-025-02789-w (PMC11796080; doi:10.1186/s12893-025-02789-w)
Supplement: Supplementary file 4 — Supplementary Material 4 [file 12893_2025_2789_MOESM4_ESM.docx]

**Additional File 4**

**Aggregation of the Perceptions of the Time-out Variable**

To account for the effect of sampling error, we constructed confidence intervals around each team's agreement index for each variable of interest. This approach was chosen in light of ongoing debates in the literature regarding the appropriateness of strict threshold criteria, such as the .7 RWG cutoff.

We used bootstrapping (50) to create many simulated samples from the observed data, which were used to obtain a confidence interval of r_wg_for each team and variable (51). The following steps were taken to compute the r_wg_: the responses to the questionnaire items were averaged per individual. 1,000 bootstrap replications were created with the data by sampling the sample data size with replacement. We computed r_wg_ for each team on each bootstrapped sample for each construct.  Confidence intervals were obtained for each team’s r_wg_ per construct using percentile cutoffs of 2.5% and 97.5% for the observed r_wg_ values across the 1,000 replications. We examined whether the upper bound of the confidence intervals included .7, which is a commonly used cut-off score (39). This was true for all teams and variables; therefore, the individual averages for each construct were aggregated to the team level and all further analysis was computed with team-level variables. Table S4 provides the details on the Rwg and the confidence intervals for each team for the perceived utility of the time-out variable.

**Table S4**

*R_wg_ and Confidence Interval for the Perceived Utility of the Time-Out Variable*

| Team | RWG | | Confidence Interval | | |
| --- | --- | --- | --- | --- | --- |
|  | Mean | St Dev | 2.50% | 50% | 97.50% |
| 1 | 0.631 | 0.236 | 0.21830357 | 0.625 | 0.9375 |
| 2 | 0.893 | 0.067 | 0.83333333 | 0.89880952 | 1 |
| 3 | 0.304 | 0.513 | 0 | 0.07013889 | 0.9166667 |
| 4 | 0.279 | 0.489 | 0 | 0 | 1 |
| 5 | 0.631 | 0.286 | 0.16666667 | 0.5 | 0.9583333 |
| 6 | 0.658 | 0.141 | 0.33333333 | 0.46428571 | 1 |
| 7 | 0.643 | 0.303 | 0.52743056 | 0.79166667 | 0.968936 |
| 8 | 0.717 | 0.118 | 0.125 | 0.55208333 | 0.9583333 |
| 9 | 0.696 | 0.261 | 0.21525298 | 0.62808442 | 0.95 |
| 10 | 0.929 | 0 | 0.7140625 | 0.85 | 0.9821429 |
| 11 | 0.565 | 0.143 | 0 | 0.45 | 0.9628571 |
| 12 | 0.488 | 0.202 | 0 | 0.37547348 | 0.8809524 |
| 13 | 0.524 | 0.118 | 0 | 0.41527778 | 0.8933036 |
| 14 | 0.863 | 0.025 | 0.6125 | 0.85 | 0.9791667 |
| 15 | 0.529 | 0.136 | 0 | 0.55 | 0.9641667 |
| 16 | 0.018 | 0.008 | 0 | 0 | 0.9640625 |
| 17 | 0.476 | 0.404 | 0.20833333 | 0.55208333 | 0.9583333 |
| 18 | 0.804 | 0.194 | 0.70833333 | 0.82954545 | 0.9795573 |
| 19 | 0.78 | 0.025 | 0.46817708 | 0.71875 | 0.975 |
| 20 | 0.47 | 0.53 | 0.12222222 | 0.484375 | 0.9285714 |
| 21 | 0.714 | 0.118 | 0.33333333 | 0.65228175 | 0.9166667 |
| 22 | 0.673 | 0.059 | 0.35 | 0.6875 | 0.975 |
| 23 | 0.518 | 0.227 | 0 | 0.4875 | 0.8435227 |
| 24 | 0.595 | 0.354 | 0.30208333 | 0.5952381 | 0.96875 |
| 25 | 0.982 | 0.025 | 0.9375 | 0.98452381 | 1 |
| 26 | 0.192 | 0.471 | 0 | 0 | 0.9583333 |
| 27 | 0.702 | 0.337 | 0.16625 | 0.7 | 0.984375 |
| 28 | 0.857 | 0.118 | 0.68727679 | 0.81628788 | 0.9708333 |
| 29 | 0.798 | 0.118 | 0.20833333 | 0.69412879 | 0.975 |
| 30 | 0.506 | 0.177 | 0 | 0.25 | 0.9405195 |
| 31 | 0.935 | 0.008 | 0.83333333 | 0.9375 | 0.984375 |
| 32 | 0.467 | 0.66 | 0.0953125 | 0.54861111 | 0.96875 |
| 33 | 0.467 | 0.66 | 0 | 0.55902778 | 0.9619792 |
| 34 | 0.713 | 0.159 | 0.33333333 | 0.55448718 | 1 |
| 35 | 0.929 | 0 | 0.83333333 | 0.88333333 | 0.96875 |
| 36 | 0.887 | 0.059 | 0.69754464 | 0.8125 | 0.9583333 |
| 37 | 0.762 | 0.118 | 0.71666667 | 0.84151786 | 0.9703869 |
| 38 | 0.863 | 0.025 | 0.50189732 | 0.81309524 | 0.9732143 |
| 39 | 0.548 | 0.118 | 0 | 0.46527778 | 0.96875 |
| 40 | 0.804 | 0.177 | 0.46875 | 0.77777778 | 1 |
| 41 | 0.708 | 0.059 | 0.07078598 | 0.52916667 | 0.9583333 |
| 42 | 0.256 | 0.059 | 0 | 0.21643201 | 0.7858631 |
| 43 | 0.649 | 0.093 | 0.18166667 | 0.62916667 | 0.9133185 |
| 44 | 0.81 | 0.168 | 0.4 | 0.72321429 | 1 |
| 45 | 0.783 | 0.212 | 0.33333333 | 0.69047619 | 1 |
| 46 | 0.783 | 0.212 | 0.33333333 | 0.69198232 | 1 |
| 47 | 0.786 | 0.017 | 0.33333333 | 0.69866071 | 0.9583333 |
| 48 | 0.508 | 0.212 | 0 | 0.29694264 | 0.9245536 |
| 49 | 0.095 | 0.118 | 0 | 0 | 0.9286558 |
| 50 | 0.845 | 0.135 | 0.55208333 | 0.8125 | 0.975 |
| 51 | 0.714 | 0.354 | 0.3310119 | 0.71428571 | 1 |
| 52 | 0.399 | 0.177 | 0 | 0.23809524 | 0.9666667 |
| 53 | 0.756 | 0.227 | 0.525 | 0.72321429 | 0.9308842 |
| 54 | 0.762 | 0.135 | 0.51645833 | 0.775 | 0.9404978 |
| 55 | 0.661 | 0.076 | 0 | 0.5 | 0.9375 |
| 56 | 0.685 | 0.008 | 0.22916667 | 0.5625 | 0.8936932 |
| 57 | 0.673 | 0.244 | 0.2125 | 0.60825893 | 0.9750868 |
| 58 | 0.399 | 0.177 | 0 | 0.359375 | 0.9166667 |
| 59 | 0.863 | 0.025 | 0.71875 | 0.9 | 1 |
| 60 | 0.813 | 0.265 | 0.55357143 | 0.8125 | 1 |
