# Supplementary material for: The surgical time-out: the relationship between perceptions of a safety-task anchor and surgical team workflow
Source: BMC Surg. 2025 Feb 5;25:55. doi: 10.1186/s12893-025-02789-w (PMC11796080; doi:10.1186/s12893-025-02789-w)
Supplement: Supplementary file 5 — Supplementary Material 5 [file 12893_2025_2789_MOESM5_ESM.docx]

**Additional File 5**

**Control Analysis**

To gain an understanding of which variables would be affected by the different surgeons present during the surgical procedures (Table S5) and the location where the data was collected (Table S6) the following analysis were performed.

**Table S5**

*Overview of Control Analysis Involving Surgeons*

| Surgeon in relation to: | Homogeneity of variance | Multiple comparison test | Control |
| --- | --- | --- | --- |
| Length of time-out | Significant | Dunnett | Yes |
| Nurse leaving OR | Non-significant | ANOVA with Tukey HSD | No |
| Perception of the time-out | Non-significant | ANOVA with Tukey HSD | No |

**Surgeons and Length of the Time-Out.** We tested for homogeneity of variance across surgeons in regards to length of the time-out. The test for homogeneity was significant, thereby not meeting the assumption of homogeneity of variance. A Dunnet test was conducted to understand which surgeons differ from one another in terms of length of the time-out. The results showed that multiple surgeons differ from one another.

To control for the surgeon in regard to length of the time-out, we performed a regression with the dummy coded surgeons as independent variables and the length of the time-out as the dependent variable. Through the regression we obtained the standardized residuals of the length of the time-out based on which surgeon led the surgical procedure. The adjusted measure was used in the model.

**Surgeons and Frequency with Which the Circulating Nurse Leaves the OR.** The frequency with which the circulating nurse leaves the OR was controlled for by the length of surgery. We tested for homogeneity of variance across surgeons in regards to the adjusted frequency with which the circulating nurse left the OR. The test for homogeneity was non-significant. We conducted a one-way ANOVA with a Tukey HSD test to determine if there were any significant differences among surgeons in relation to the frequency with which the circulating nurse left the OR. The results showed no significant differences and therefore we did not control for surgeon when involving the measure of frequency of the nurse leaving the OR.

**Surgeons and Perceptions of the Time-Out.** We tested for homogeneity of variance across surgeons in regard to perceptions of the time-out. The test for homogeneity was non-significant. We conducted a one-way ANOVA with a Tukey HSD test to determine if there were any significant differences among surgeons in relation to the perceptions of the time-out. The results showed no significant differences; therefore, we did not control for surgeon when involving perceptions of the time-out.

**Differences Among Locations in Which the Data Was Collected.** The data was collected in two sites. To determine if there were any differences among the hospitals, we conducted a one-way analysis of variance with perceptions of the time-out, length of the time-out, and frequency with which the nurse leaves the OR as the dependent variables and location as the independent variable. Table S6 shows the result of the one-way ANOVA.

**Table S6**

*One-way Analysis of Variance for Location in which the Data was Collected*

| Dependent Variable | F statistic | Significance |
| --- | --- | --- |
| Perceptions of the time-out | .507 | .479 |
| Length of time-out | 5.85 | .019 |
| Frequency of nurse leaving | .175 | .677 |

Note. The frequency of nurse leaving have been controlled for length of surgery.

The results of the one-way ANOVA show that there are statistically significant differences in terms of location for the length of the time-out. To control for the location in regard to length of the time-out, we performed a regression with the dummy coded location as independent variables and the length of the time-out (already adjusted for surgeon) as the dependent variables. Through the regression, we obtained the standardized residuals of the length of the time-out adjusted for location (and surgeon).
